# Mutations in the microRNA172 binding site of *SUPERNUMERARY BRACT (SNB)* suppress internode elongation in rice

**DOI:** 10.1186/s12284-019-0324-8

**Published:** 2019-08-09

**Authors:** Hyeonso Ji, Chang-deok Han, Gang-Seob Lee, Ki-Hong Jung, Do-Yu Kang, Jun Oh, Hyoja Oh, Kyeong-Seong Cheon, Song Lim Kim, Inchan Choi, Jeongho Baek, Kyung-Hwan Kim

**Affiliations:** 1 Department of Agricultural Biotechnology, National Institute of Agricultural Sciences (NAS), Jeonju, 54874 South Korea; 2 Division of Applied Life Science (BK21 Program), Plant Molecular Biology and Biotechnology Research Center (PMBBRC), Gyeongsang National University, Jinju, 52828 South Korea; 3 The Graduate School of Biotechnology and Crop Biotech Institute, Kyung Hee University, Yongin, 17104 South Korea

**Keywords:** Rice, Internode elongation, SNB, microRNA172

## Abstract

**Background:**

Internode elongation is an important agronomic trait in rice that determines culm length, which is related to lodging, panicle exsertion, and biomass. *sui4* (*shortened uppermost internode 4*) mutants show reduced internode length and a dwarf phenotype due to shortened internodes; the uppermost internode is particularly severely affected. The present study was performed to identify the molecular nature and function of the *SUI4* gene during internode elongation.

**Results:**

Our previous study showed that the *SUI4* gene was mapped to a 1.1-Mb interval on chromosome 7 (Ji et al. 2014). In order to isolate the gene responsible for the *sui4* phenotype, genomic DNA resequencing of *sui4* mutants and wild-type plants and reciprocal transformation of wild-type and mutant alleles of the putative *SUI4* gene was performed. The data revealed that the causative mutation of *sui4* was a T to A nucleotide substitution at the microRNA172 binding site of *Os07g0235800*, and that *SUI4* is a new allele of the previously reported gene S*UPERNUMERARY BRACT (SNB),* which affects flower structure. In order to understand the effect of this mutation on expression of the *SUI4/SNB* gene, *SUI4*/*SNB* native promoter-fuzed GUS transgenics were examined, along with qRT-PCR analysis at various developmental stages. In *sui4* mutants, the *SUI4/SNB* gene was upregulated in the leaves, culms, and panicles, especially when internodes were elongated. In culms, *SUI4/SNB* was expressed in the nodes and the lower parts of elongating internodes. In order to further explore the molecular nature of *SUI4*/*SNB* during internode elongation, RNA-seq and qRT-PCR analysis were performed with RNAs from the culms of *sui4* mutants and wild-type plants in the booting stage. The data showed that in *sui4* mutants, genes deactivating bioactive gibberellins and cytokinin were upregulated while genes related to cell expansion and cell wall synthesis were downregulated.

**Conclusion:**

In summary, this paper shows that interaction between *SUI4*/*SNB* and microRNA172 could determine internode elongation during the reproductive stage in rice plants. Due to a mutation at the *microRNA172* binding site in *sui4* mutants, the expression of *SUI4*/*SNB* was enhanced, which lowered the activities of cell expansion and cell wall synthesis and consequently resulted in shortened internodes.

**Electronic supplementary material:**

The online version of this article (10.1186/s12284-019-0324-8) contains supplementary material, which is available to authorized users.

## Background

The culm of rice plants is composed of solid nodes and hollow internodes. Internode elongation is an important agronomic trait that determines culm length, which is related to lodging, panicle exsertion, and biomass. Rice internodes can be divided into three regions: the intercalary meristem (IM), the elongation zone (EZ), and the differentiation zone (DZ) (Kende et al. 1998). At the base of the internode is the IM, where cell division generates new internodal cells. These new cells are displaced into the EZ, where they elongate until they reach their final length, and growth ceases in the DZ, where formation of the secondary wall and xylem takes place (Kende et al. 1998).

Gibberellin (GA) activates the cell cycle in the IM as well as cell elongation (Kende et al. 1998). Mutations in genes involved in GA biosynthesis and signaling hamper internode elongation and cause dwarfism, as exemplified in rice *sd1, d18, d35, d1, gid1,* and *gid2* mutants. GA signals promote cellulose synthesis by preventing interaction between SLENDER RICE1 (SLR1), a DELLA repressor of GA signaling, and NACs, the top-layer transcription factors for secondary wall formation (Huang et al. 2015).

Brassinosteroids (BRs) are also essential in stem elongation (Je et al. 2010). Under physiological conditions, BRs promote GA accumulation by regulating the expression of GA metabolic genes to stimulate cell elongation (Tong et al. 2014). BRs increase the expression of two bHLH transcription factors, *ILI1 (INCREASED LAMINA INCLINATION 1)* and *PRE1 (PACLOBUTRAZOL RESISTANCE 1),* which both increase cell elongation, but repress the bHLH transcription factor *IBH1 (ILI1 BINDING BHLH 1)* through *BZR1 (BRASSINAZOLE-RESISTANT 1)*, which suppresses cell elongation (Zhang et al. 2009). Several rice dwarf mutants had mutations in genes involved in BR biosynthesis and signaling; they are *d2*, *d11*, *brd1, brd2, d61,* and *dlt.*

The main culm of japonica rice has 14–17 nodes; internode elongation occurs only above the fifth node from the top, while the lower internodes remain unelongated (Chonan 1993). The uppermost internode is the longest, and lower internodes become progressively shorter (Chonan 1993). Upon shifting from vegetative to reproductive growth, four or five upper internodes (UPIs) sequentially elongate from the basal end to the uppermost internode. This process is concomitant with panicle development, suggesting that signaling occurs between panicles and UPIs during the reproductive phase (Itoh et al. 2005; Wang et al. 2009; Yamamuro et al. 2000). Identification of regulators of UPI elongation and floral development should provide clues as to the underlying genetic factors that coordinate organogenesis in both vegetative phytomers and panicles (Wang et al. 2009).

microRNAs play important roles in controlling plant development, productivity, and defense against biotic and abiotic stresses by negatively regulating gene expression at the post-transcriptional level (Teotia and Tang 2015). miR172 regulates the expression of a small group of AP2-like transcription factors and the transitions between developmental stages, as well as functioning in the specification of floral organ identity (Zhu and Helliwell 2011). Rice miR172 induces flowering by suppressing *OsIDS1 (Oryza sativa INDETERMINATE SPIKELET1)* and *SNB (SUPERNUMERARY BRACT)*, two AP2 genes that negatively regulate the expression of *Ehd1 (EARLY HEADING DATE 1)* (Lee et al. 2014). The *SNB* gene, which is one of targets of miR172, controls the transition from spikelet meristem to floral meristem; in *SNB* knockout plants, the transition from spikelet meristems to floral meristems is delayed, resulting in the production of multiple rudimentary glumes (Lee et al. 2007). Overexpression of miR172b in rice has been shown to delay the transition from spikelet meristem to floral meristem, resulting in floral and seed developmental defects including changes in the number and identity of floral organs (Zhu et al. 2009).

In our previous study, we characterized *sui4* mutant rice plants (Ji et al. 2014). The uppermost internodes of the *sui4* mutants were severely shortened, which caused enclosure of panicles in the flag leaf sheath. The cells in these uppermost internodes were also remarkably shorter and more densely packed than those in the wild type (Dongjin). The *SUI4* gene showed incomplete dominance or semidominance and was mapped to a 1.1-Mb region, *RM1253-S0701579,* on chromosome 7.

In the present study, we identified the molecular nature of the *sui4* mutation by resequencing of mutant and wild-type genomic DNA and by reciprocal transformation of *SUI4* alleles. It was revealed that a single nucleotide substitution in the miR172 binding site resulted in the dwarf phenotype and that *SUI4* was allelic to *SNB*. To understand the effect of the mutation on expression of the *SUI4/SNB* gene, *SUI4*/*SNB* native promoter-fuzed GUS transgenics and qRT-PCR were employed. RNA-seq analysis demonstrated that *SUI4/SNB* could affect the homeostasis of GA and cytokinin. This study is the first to show that the expression interaction between the AP2 domain gene and miR172 determines the elongation of upper internodes in rice.

## Results

### A point mutation in *SUI4* causes upregulation of this gene by blocking miRNA172 binding

**Fig. 1:**
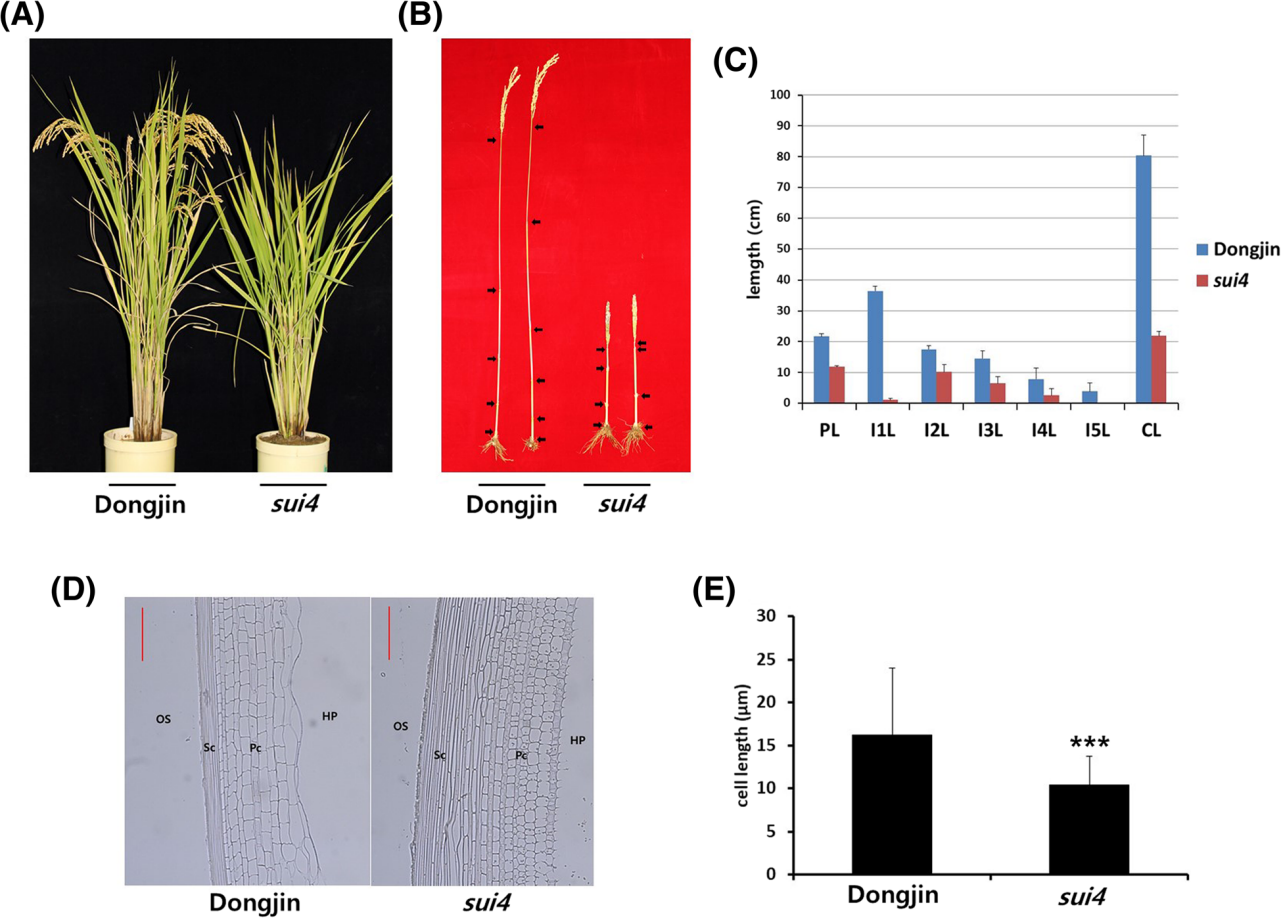
Phenotype comparison between Dongjin plants and *sui4* mutants. (**a**) Plant architecture at maturing stage. (**b**) Culm structure. Arrows indicate locations of nodes. Two main culms from two plants are shown for each phenotype. (**c**) Comparison of internode length between Dongjin plants and *sui4* mutants. PL: panicle length, I1L: first internode length, I2L: second internode length, I3L: third internode length, I4L: fourth internode length, CL: culm length. (**d**) Longitudinal sections of the uppermost internodes of Dongjin plants and *sui4* mutants. Red bars denote 50 μm. HP: hollow pith, OS: outer space, Sc: sclerenchyma tissue, Pc: parenchyma tissue. (**e**) Cell length in the uppermost internodes of Dongjin plants and *sui4* mutants. The lengths of cells in parenchyma tissues were measured. Data are shown as the mean ± s.d. (t-test, *** *P* < 0.001). The cells in three samples were measured for Dongjin plants and *sui4* mutants, respectively

Relative to wild-type (Dongjin) plants, *sui4* mutants showed reduced internode length and eventually a dwarf phenotype. In the mutants, the uppermost internode was much more severely shortened than the lower internodes (Fig.1a, b, c). The length of individual cells in the uppermost internode was also much shorter in *sui4* mutants than in Dongjin plants (Fig. 1d, e).

**Fig. 2:**
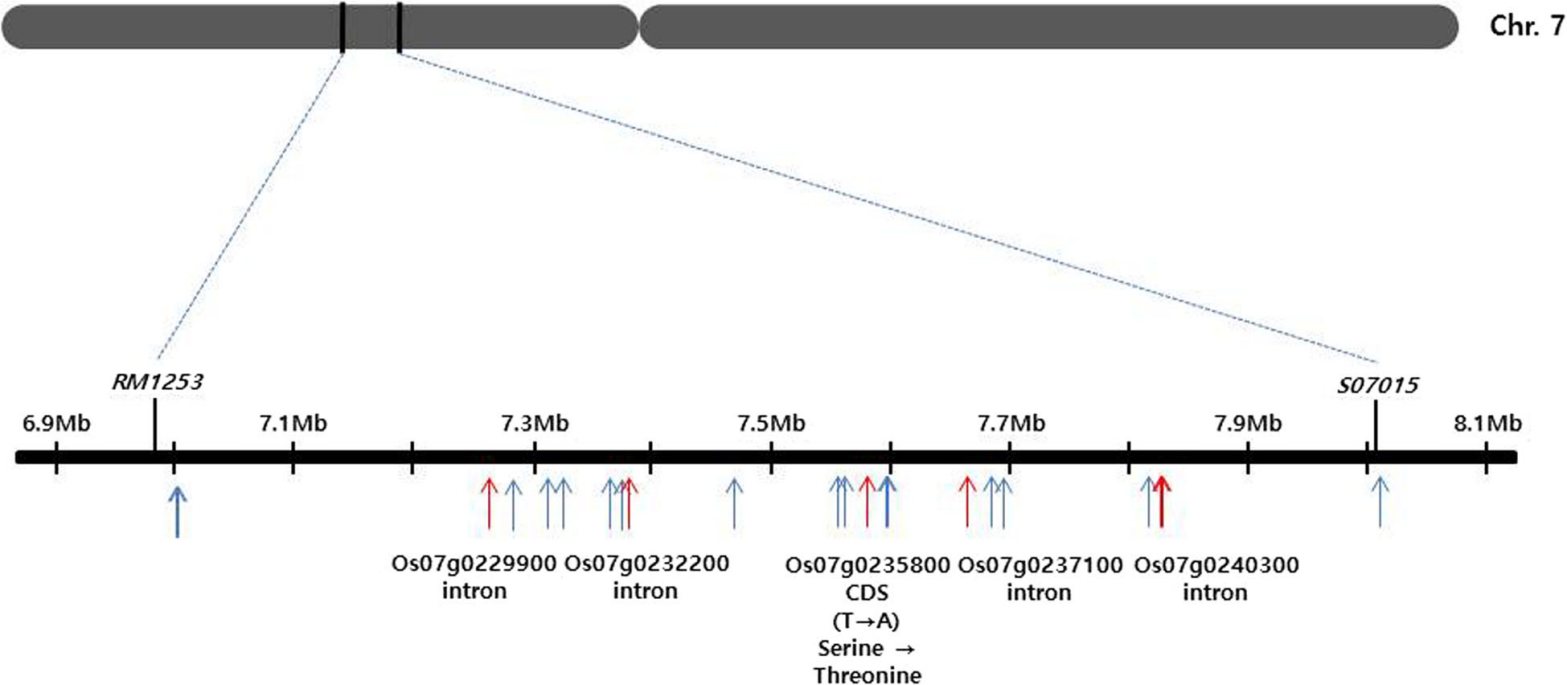
SNPs found in the *SUI4* gene region on chromosome 7. Twenty-two SNPs were identified between *sui4* and Dongjin in the 1.1- Mb interval region (*RM1253-S07015*) mapped in our previous study (Ji et al. 2014). Detailed information was shown in Additional file 1: Table S1. Red arrows indicate the positions of SNPs in genic regions while blue arrows indicate the positions of SNPs in intergenic regions

To identify the locus responsible for the *sui4* phenotype, *sui4* mutants and Dongjin plants were subjected to genomic resequencing. Twenty-two SNPs were identified in the 1.1-Mb interval region mapped in our previous study (Additional file 1: Table S1, Fig. 2) (Ji et al. 2014). Only six SNPs were located in genic regions, while the rest were in intergenic regions. Among the six genic SNPs, five were located within introns, while only one was in the coding sequence of the locus *Os07g0235800*. The SNP in the coding region created a missense mutation that resulted in a single amino acid substitution from serine to threonine. The expression of five genes harboring all six genic SNPs was examined by RT-PCR. Except for *Os07g0235800*, genes carrying SNPs within introns were expressed at the same levels in Dongjin plants and *sui4* mutants (Additional file 4: Figure S1). Since the five SNPs within introns did not seem to affect expression levels, it is likely that they are not responsible for the mutation of *SUI4*. Thus, *Os07g0235800,* which was expressed at higher levels in *sui4* mutants than in Dongjin plants, was suspected to be the most probable candidate gene for the *SUI4* phenotype.

**Fig. 3:**
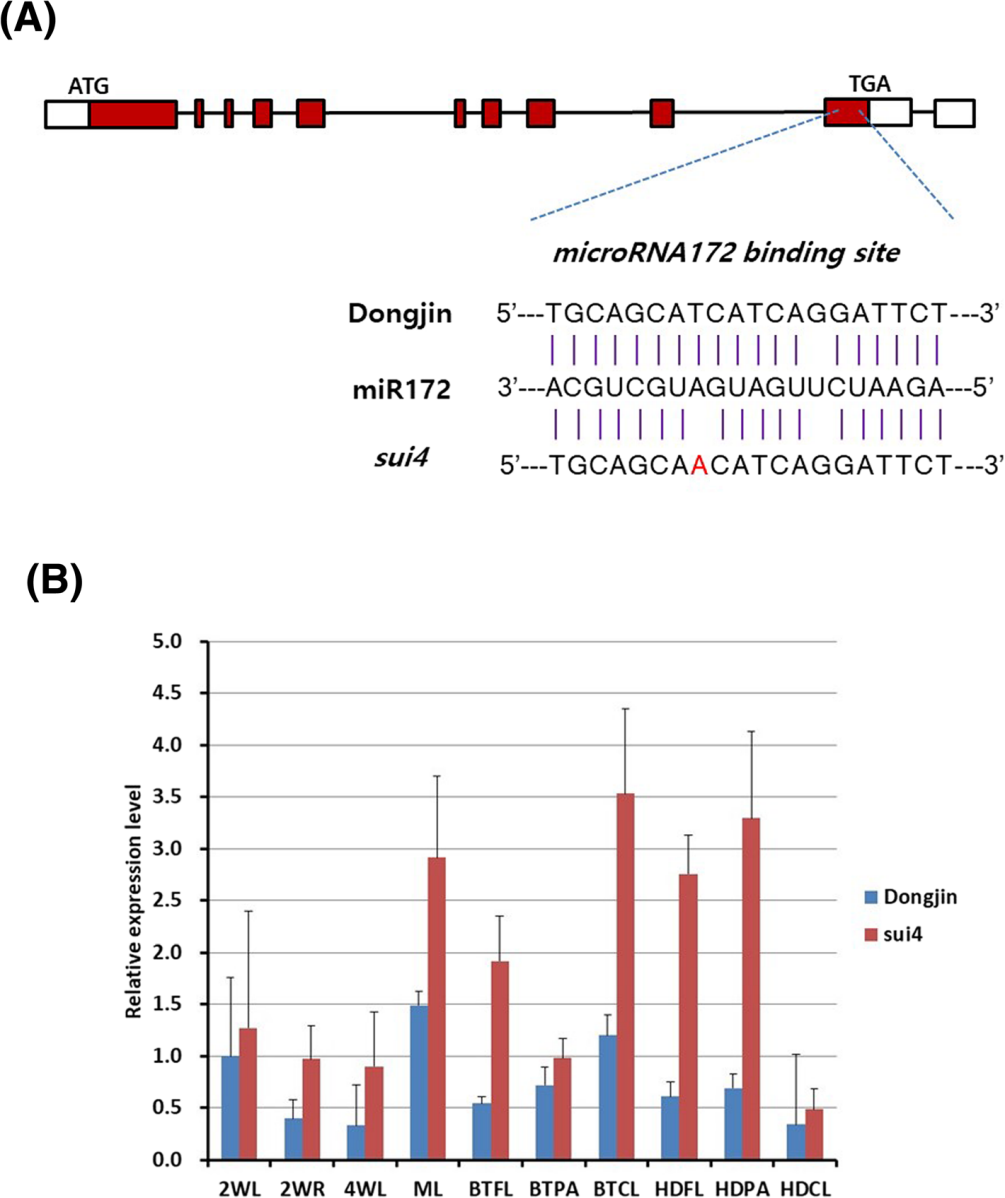
*Os07g0235800* gene mutation and expression pattern. (**a**) Structure of the *Os07g0235800* gene and location of mutation. Empty boxes indicate 5′ and 3′ UTRs, filled boxes indicate CDS, and lines indicate introns. (**b**) Expression pattern of *Os07g0235800* at different developmental stages and organs in Dongjin plants and *sui4* mutants. Relative fold expression difference is based on the expression level detected in two-week-old leaves as a baseline. Error bars represent standard deviation of the expression ratio. 2WL: two-week-old seedling leaf. 2WR: two-week-old seedling root. 4WL: four-week-old seedling leaf. ML: middle stage (70-day-old plant) leaf, BTFL: flag leaf of booting stage plant, BTPA: panicle of booting stage plant, BTCL: culm of booting stage plant, HDFL: flag leaf of heading stage plant, HDPA: panicle of heading stage plant, HDCL: culm of heading stage plant

Interestingly, the SNP was located at the miR172 binding site of *Os07g0235800*, leading to the hypothesis that the SNP might disrupt the affinity of miR172 to the binding site (Fig. 3a), which would weaken miR172-mediated suppression of *Os07g0235800*. To explore the effect of the putative miR172 binding site on the expression of *Os07g0235800*, the expression patterns of *Os07g0235800* were compared at different developmental stages (seedling, booting, and heading stages) and in different tissues (leaves, roots, and culms) between Dongjin plants and *sui4* mutants (Fig. 3b). Only in the leaves of two-week-old seedlings, booting stage panicles,and heading stage culms were the expression levels of *SUI4* similar between Dongjin plants and *sui4* mutants. In all other samples, levels of *SUI4* mRNA were higher in *sui4* mutants than in Dongjin plants. Expression was particularly high in *sui4* mutant culms in the booting stage, when the culm was growing rapidly. Meanwhile, during the heading stage, when culm growth ceased, *SUI4* expression was higher in *sui4* mutant flag leaves and panicles than in culms. In Dongjin plants, the highest level of *SUI4* mRNA expression was in the leaves of 70-day-old plants. This comparative expression study indicates that the expression of *Os07g0235800* in Dongjin plants might be affected by the miR172 binding site, which, in *sui4* mutants*,* harbored a point mutation. Overall, the difference in *Os07g0235800* expression between Dongjin plants and *sui4* mutants became more pronounced in the later stages of development, when the culm was growing rapidly. This is in agreement with previous reports of microRNA172 expression increasing as plants grow (Zhu and Helliwell 2011; Zhu et al. 2009).

**Fig. 4:**
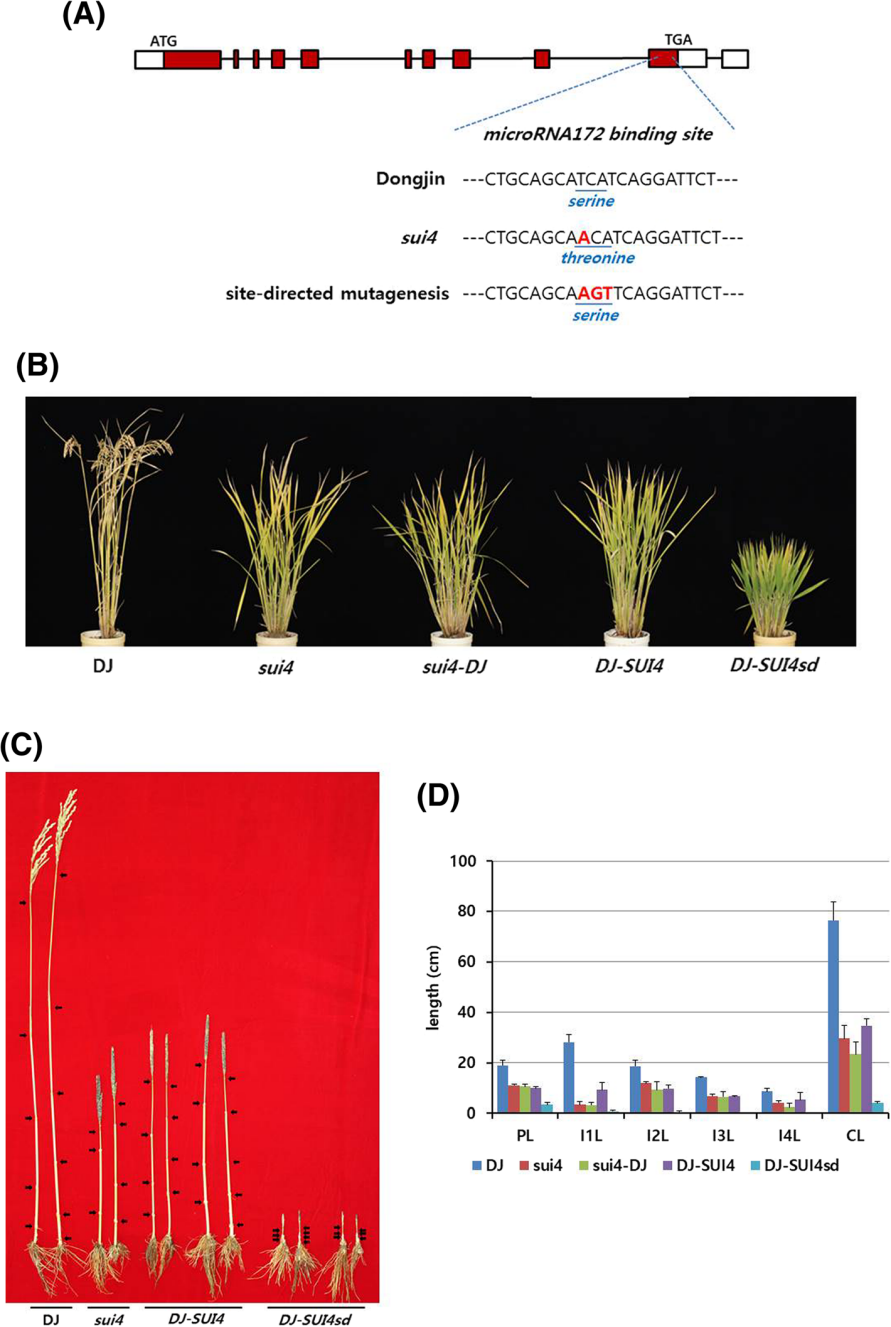
Mutations in the *Os07g0235800* gene and phenotypes of transgenic plants. (**a**) Structure of the *SUI4/SNB* gene and location of mutations. Empty boxes indicate 5′ and 3′ UTRs, filled boxes indicate CDS, and lines indicates introns. (**b**) Plant architecture at the maturing stage. DJ: Dongjin, *sui4-DJ*: transgenic line in which the *SUI4* gene from a Dongjin plant was introduced into *sui4* mutants, *DJ-SUI4*: transgenic line in which the *SUI4* gene from a *sui4* mutant was introduced into Dongjin plants, *DJ-SUI4sd*: transgenic line in which the *SUI4* gene, modified by site-directed mutagenesis as shown in (**a**), was introduced into Dongjin plants. (**c**) Culm structure. Arrows indicate locations of nodes. Two main culms from two plants are shown for each phenotype. Two independent transgenic lines are shown for *DJ-SUI4* and *DJ-SUI4sd.* (**d**) Lengths of panicles, culms, and internodes. For DJ and *sui4*, three main culms from three different plants were measured. For *sui4*-*DJ*, *DJ-SUI4*, and *DJ-SUI4sd*, six main culms from three to four independent transgenic lines were measured. PL: panicle length, I1L: first internode length, I2L: second internode length, I3L: third internode length, I4L: forth internode length, CL: culm length

In order to clarify the genetic relationship between the wild-type *SUI4* allele of Dongjin plants and the *SUI4* allele of the *sui4* mutant, genomic DNA containing *Os07g0235800* was isolated from Dongjin plants and *sui4* mutants and was reciprocally transformed into *sui4* mutants and Dongjin plants, respectively. First, a vector harboring the 7.9-kb genomic DNA that contained the genic region of wild-type *Os07g0235800,* including its promoter and terminator, was transformed from a Dongjin plant into *sui4* mutants. The resulting transgenic line, referred to as *sui4-DJ*, was not significantly different from *sui4* mutants (Fig. 4a-d, Additional file 5: Figure S2). This may be due to the fact that *SUI4* shows semi-dominant characteristics (Ji et al. 2014). In contrast, transformation of a vector harboring a 7.9-kb genic region of *Os07g0235800,* including its promoter and terminator, from a *sui4* mutant into Dongjin plants caused a remarkable reduction in internode length in the resulting transgenic line, *DJ*-*SUI4* (Fig. 4a–d, Additional file 5: Figure S2). This demonstrates that *Os07g0235800* is responsible for the *sui4* phenotype.

Next, since *sui4* mutants harbored the single T to A nucleotide substitution, which resulted in one amino acid substitution from serine (TCA) to threonine (ACA), an experiment was performed to determine whether the mutant phenotype arose due to a single amino acid substitution or due to the single nucleotide change in the miR172 binding site. A vector was constructed in which the amino acid threonine (ACA) of *sui4* was changed to the original amino acid serine (AGT) by substituting CA with GT, which resulted in three nucleotide changes in the original microRNA172 binding site but retained the original amino acid sequence (Fig. 4a). The vector was transformed into Dongjin plants, which produced the transgenic line *DJ-SUI4sd*. The transgenic lines showed more severe dwarf phenotypes and much more severely reduced internode lengths than *sui4* mutants (Fig. 4b-d). Therefore, the amino acids encoded by the microRNA172 binding site are not related to phenotypic expression. The observation that *DJ-SUI4sd* plants exhibited a stronger phenotype than *sui4* mutants indicates that the strength of the mRNA binding site affinity could determine phenotypic expression. The expression of *Os07g0235800* in leaves from 14, 28, and 77 days old plants of Dongjin, *sui4*, *DJ-SUI4*, and *DJ-SUI4sd* plants were measured by qRT-PCR (Additional file 7: Figure S4). Dongjin showed the lowest expression while *DJ-SUI4*, and *DJ-SUI4sd* plants showed higher expression than Dongjin and *sui4* plants.

Taken together, these results demonstrate that interruption of the affinity of miR172 to the binding site of *Os07g0235800* was the cause of reduction in internode length. Interestingly, *Os07g0235800* has been reported to be the *SNB* gene, which controls the transition from spikelet meristem to floral meristem and floral organ development (Lee et al. 2007). Therefore, it was concluded that *SUI4* and *SNB* are the same gene, controlling both internode elongation and floral development during the reproductive stage.

### Analysis of the expression pattern of *SUI4*/*SNB* using promoter-GUS systems

**Fig. 5:**
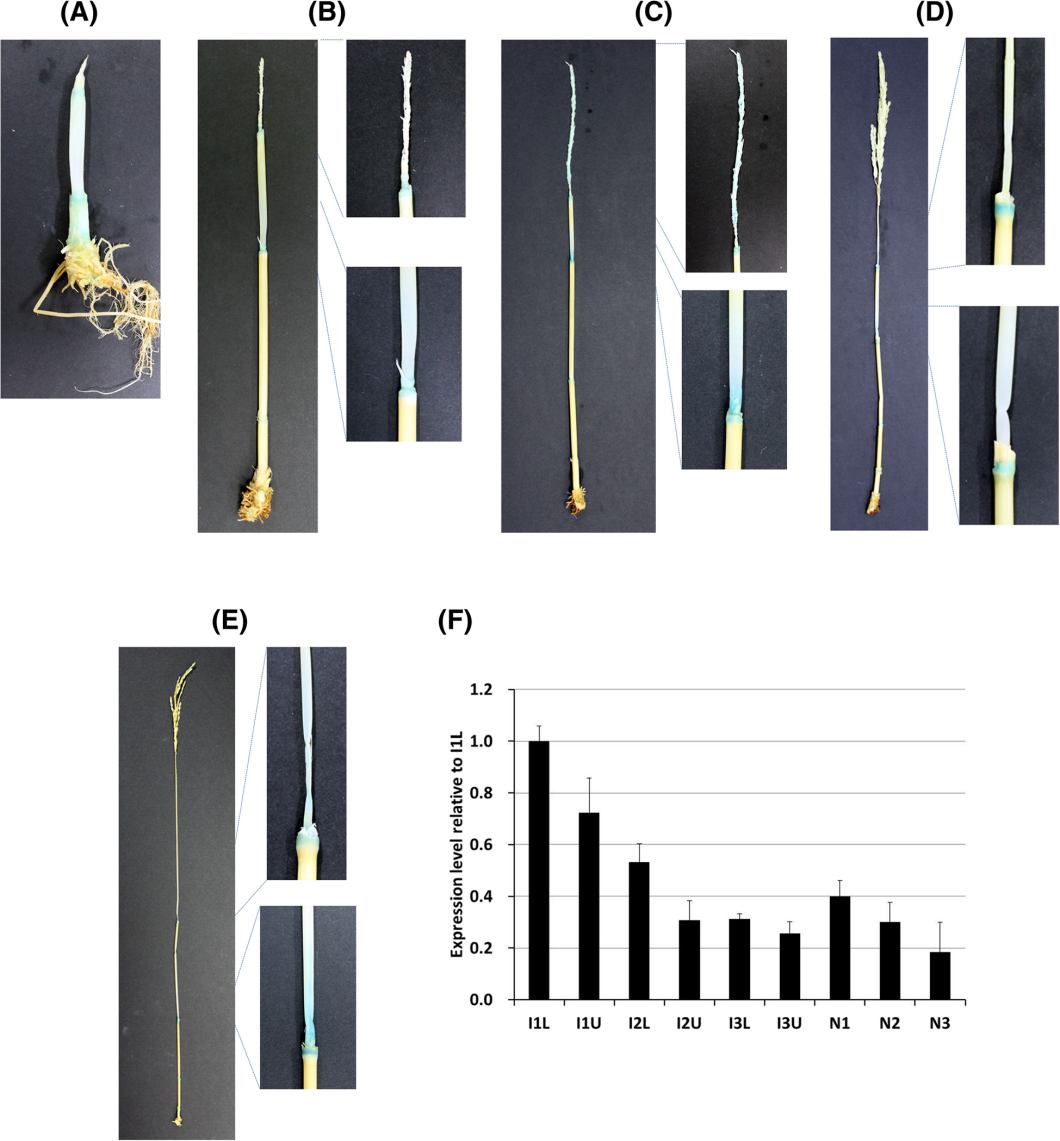
GUS expression pattern of SNB promoter:GUS fusion gene transgenic lines. (**a**) Culm of rice plant when panicle length was 0.6 cm. (**b**) Culm of rice plant when panicle length was 3.5 cm. (**c**) Culm of rice plant when panicle length was 12.4 cm. (**d**) Culm of rice plant when panicle length was 17.7 cm. (**e**) Culm of rice plant when panicle length was 20.8 cm (heading stage). (**f**) Results of qRT-PCR analysis of the *SUI4* gene in the culm of a heading stage plant. I1L: lower half of the first internode, I1U: upper half of the first internode, I2L: lower half of the second internode, I2U: upper half of the second internode, I3L: lower half of the third internode, I3U: upper half of the third internode, N1: first node, N2: second node, N3: third node

Since previous work on *Os07g0235800* as *SNB* has focused on floral organs or panicles (Lee et al. 2007), it was necessary to examine the expression of *Os07g0235800* during the growth of culms. In order to obtain information on spatial and temporal gene expression patterns, a fusion construct between the 2-kb *SUI4/SNB* promoter and the *GUS* reporter gene was introduced into Dongjin plants. In the month prior to the heading stage, when panicles were developed and internodes became elongated, culms of transgenic plants were taken weekly to analyze GUS expression patterns. When panicle length reached 0.6 cm, GUS expression was observed in nodes and the lower parts of internodes where internodes were expected to be elongated (Fig. 5a). In plants with young panicles of 3.5 cm, GUS expression was observed in nodes and the lower part of the uppermost internode. In contrast, little GUS expression was detected in the lower internodes that ceased to elongate (Fig. 5b). When the panicle length reached 12.4 cm, GUS expression was observed in the panicles, nodes, and lower parts of the uppermost internode, but there was little GUS expression in the lower internodes that had ceased elongation (Fig. 5c). When panicle length reached 17.7 cm, GUS expression was observed in nodes and the lower part of elongating upper internodes, but there was little GUS expression in lower internodes (Fig. 5d). During the heading stage, GUS expression was observed in nodes and the lower parts of the two uppermost internodes, but there was little GUS expression in the lower internodes (Fig. 5e).

Additionally, qRT-PCR was performed to verify the expression of *SUI4/SNB* in culms in the heading stage. The qRT-PCR data confirmed that expression was higher in upper internodes than in lower internodes, and that within each internode, lower parts showed higher expression than upper parts (Fig. 5f). Overall, *SUI4/SNB* was expressed in nodes and the lower part of elongating internodes in culms. These expression patterns are consistent with the idea that *SUI4/SNB* controls internode elongation.

### Transcriptome analysis of *sui4* mutants vs wild-type Dongjin plants suggests the inactivation of GA and cytokinin

**Fig. 6:**
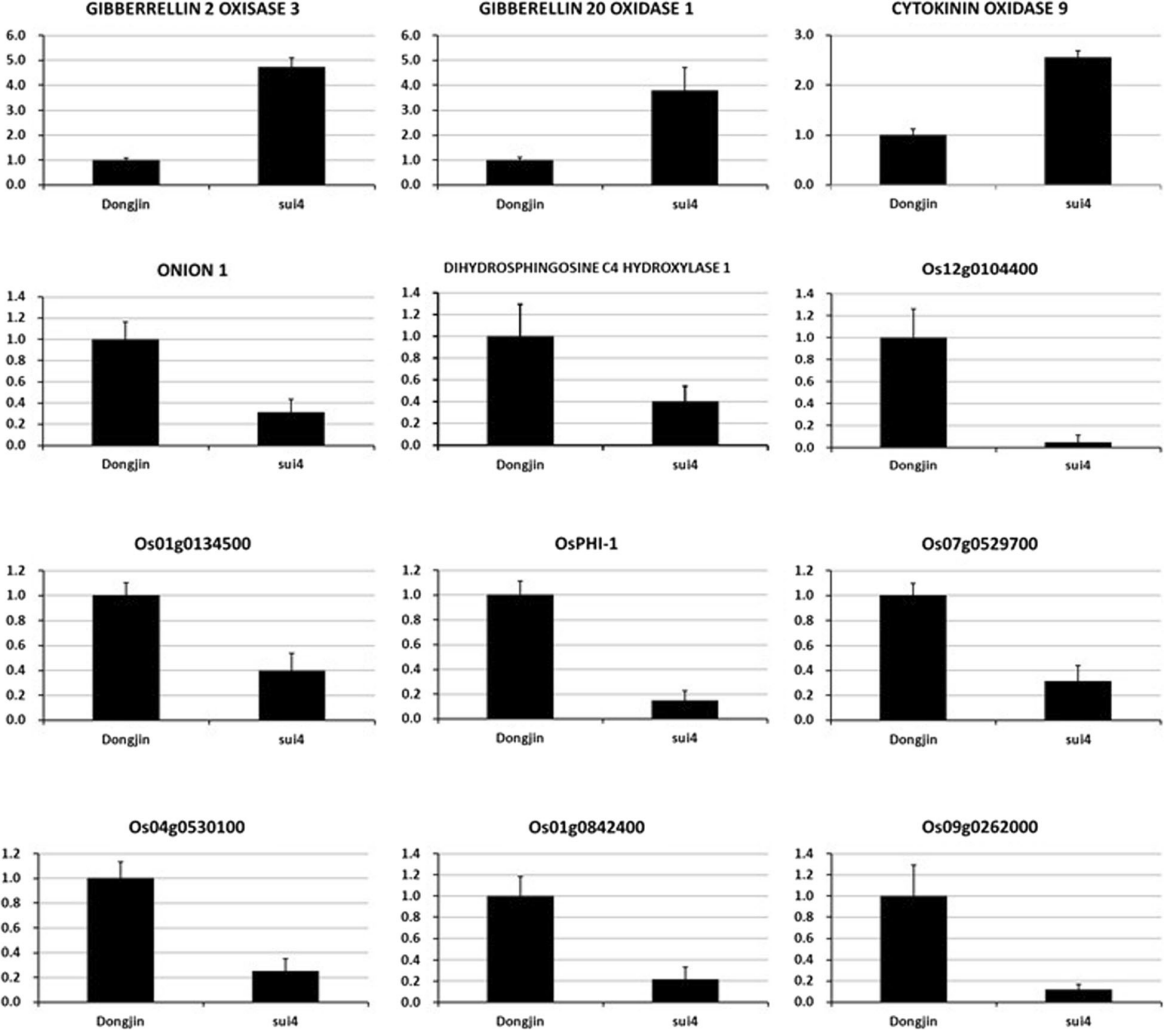
Relative expression of representative DEGs in the culm of booting stage plants, as revealed by qRT-PCR. RNA was extracted from main culms of *sui4* mutant and Dongjin palnts taken at the booting stage. The expression level in Dongjin plants was set as 1. Three biological replicates were performed. Error bars show SE (*n* = 3)

To explore the molecular effects of *SUI4/SNB* on culm elongation, RNA-seq analysis was carried out with culms in the booting stage. A bioinformatic study was performed to identify differentially expressed genes (DEGs) between *sui4* mutants and wild-type (Dongjin) plants. Compared with Dongjin plants, a total of 217 genes were found to be upregulated while 405 genes were downregulated in *sui4* mutants (≥ two-fold change; *P* < 0.05; Additional file 2: Table S2). These DEGs were subjected to gene ontology (GO) enrichment analysis. In terms of biological processes, carbohydrate biosynthesis, polysaccharide biosynthesis, glucan biosynthesis, cellular polysaccharide metabolism, and polysaccharide metabolism were significantly influenced by *SUI4/SNB* (Table 1). Representative DEGs likely to be related to the *sui4* phenotype were selected and their expression in booting stage culms was measured by qRT-PCR (Fig. 6). *GIBBERELLIN 2-OXIDASE 3,* which inactivates endogenous bioactive gibberellins (GA1, 4) (Lo et al. 2008), was markedly upregulated in *sui4* mutants*.* The depletion of bioactive GAs by increased expression of *GIBBERELLIN 2-OXIDASE 3* could be one of the major causes of internode length reduction in *sui4* mutants. Also, *GIBBERELLIN 20-OXIDASE 1*, which produces inactive GA precursors (GA9, 20) (Yamaguchi 2008), was also upregulated. It is reasonable to speculate that the upregulation of *GIBBERELLIN 20-OXIDASE 1* might be due to feedback from the decrease of bioactive GAs caused by upregulation of *GIBBERELLIN 2-OXIDASE 3*. *CYTOKININ OXIDASE 9* (*OsCKX 9*), which inactivates cytokinin (Wang et al. 2014), was also upregulated in *sui4* mutants, which might reduce cell division in *sui4* internodes. The *ONION1 (ONI1*) gene which encodes a fatty acid elongase involved in the synthesis of very-long-chain fatty acids and normal shoot development in rice (Ito et al. 2011) was downregulated in *sui4* mutants. Also, *DIHYDROSPHINGOSINE C4 HYDROXYLASE 1 (DSH1)* gene which was reported to mediate biosynthesis of sphingolipid and be abundantly expressed in vascular bundles and apical meristems (Imamura et al. 2007) was downregulated in *sui4* mutants. In addition, *Os12g0104400* which was annotated as a fatty acid desaturase type 1 domain containing protein gene was downregulated. Although the roles of fatty acid desaturase in cell division and/or cell elongation have not been elucidated fully, this gene might be involved in cell membrane synthesis required for cell expansion. Moreover, *Os01g0134500* which was annotated as a delta-7-sterol-C5 desaturase was downregulated. This gene might be involved in brassinosteroid (BR) biosynthesis considering that the Arabidopsis *dwarf7* mutant was defective in delta-7-sterol-C5 desaturase gene and BR biosynthesis was hampered in this mutant (Choe et al. 1999). It was noted that *OsPHI-1*, which has been reported to be involved in cell expansion (Aya et al. 2014), was downregulated. In addition, several genes which may be related to cell wall synthesis and cell expansion were downregulated: *Os07g0529700* (xyloglucan endo-transglycosylase-like protein), *Os04g0530100* (similar to the beta-expansin 1 precursor AtEXPB1), *Os01g0842400* (similar to laccase) and *Os09g0262000* (similar to cinnamoyl CoA reductase). Xyloglucan endo-transglycosylase was proposed to do roles of cell wall loosening, assembly, strengthening and expansion (Frankova and Fry 2013), and expansins induce cell wall extension during growth (Li et al. 2003). Laccase and cinnamoyl CoA reductase are involved in biosynthesis of lignin which is predominantly deposited in the secondary cell walls (Park et al. 2017; Swetha et al. 2018). These results indicate that *SUI4/SNB* activates GA and cytokinin degradation and suppresses genes involved in cell expansion in rice culms.

**Table 1:** GO enrichment in DEGs between Dongjin plants and *sui4* mutants

Category^a^	Term	Count^b^	*P*-value^c^	Benjamini^d^
GOTERM_BP	GO:0016051: carbohydrate biosynthetic process	10	1.00E-04	0.028881
GOTERM_BP	GO:0000271: polysaccharide biosynthetic process	8	1.49E-04	0.02157
GOTERM_CC	GO:0031988: membrane-bounded vesicle	44	2.55E-04	0.017723
GOTERM_CC	GO:0016023: cytoplasmic membrane-bounded vesicle	44	2.55E-04	0.017723
GOTERM_BP	GO:0034637: cellular carbohydrate biosynthetic process	9	2.94E-04	0.028317
GOTERM_CC	GO:0031982: vesicle	44	3.76E-04	0.013082
GOTERM_CC	GO:0031410: cytoplasmic vesicle	44	3.76E-04	0.013082
GOTERM_BP	GO:0009250: glucan biosynthetic process	7	5.52E-04	0.039626
GOTERM_BP	GO:0006073: cellular glucan metabolic process	8	7.46E-04	0.042801
GOTERM_BP	GO:0033692: cellular polysxaccharide biosynthetic process	7	7.96E-04	0.038118
GOTERM_BP	GO:0044264: cellular polysaccharide metabolic process	8	9.89E-04	0.040558

^a^Database where gene set was defined. *BP*: Biological process, *CC* Cellular compartment

^b^Number of genes in the term

^c^Fisher’s exact *P*-value was adopted to measure gene enrichment in annotation terms

^d^*P*-value corrected using the Benjamini method to reduce false positives caused by multiple testing

### Modification of flower structure

Previous work investigating the effects *SNB* on flower structure was performed with knockout mutants (Lee and An 2012; Lee et al. 2007). Our study with *sui4* mutants found alterations in floral structure (Additional file 6: Figure S3): wild-type plants have two sterile lemmas, one lemma, and one palea, while the *sui4* mutant has one sterile lemma, two lemmas, and one palea. *DJ-SUI4* transgenic plants showed a flower structure similar to that of the *sui4* mutant. It has been reported that the transition from spikelet meristem to floral meristem was delayed in *SNB* knockout plants, resulting in the production of multiple rudimentary glumes (Lee et al. 2007). In *sui4* mutant and *DJ-SUI4* transgenic plants, *SNB* was upregulated. Therefore, it might be inferred that upregulation of *SNB* caused a reduction in the number of sterile lemmas and an increase in the number of lemmas.

## Discussion

In rice, the majority of the genes determining plant height via internode elongation are directly related to phytohormone GA, BR, or strigolactone biosynthetic or signaling pathways (for review, Liu et al. 2018). However, the present study showed that *SUI4/SNB* encoding a nuclear protein carrying two AP2 domains (Lee et al. 2007) reduces internode elongation by suppressing cell elongation and cell wall synthesis in the culm. Recently, *OsAP2–39* (*Oryza sativa APETALA2–39*) carrying one AP2 domain was reported to be involved in regulating plant height by controlling the expression of ABA and GA biosynthesis genes (Yaish et al. 2010). Constitutive overexpression of *OsAP2–39* under the ubiquitin promoter leads not only to short internodes but also to a low number of tillers and late flowering. Our RNA-seq data strongly suggests that *GIBBERELLIN 2-OXIDASE 3*, which inactivates endogenous bioactive gibberellins (GA1, 4), and *CYTOKININ OXIDASE 9* (*OsCKX9*), which inactivates cytokinin (Wang et al. 2014), were upregulated in *sui4* mutants. Therefore, it is reasonable to state that these AP domain genes operate regulatory mechanisms that control the hormonal homeostasis necessary for internode elongation. Notably, it was reported that the second AP2 domain of Arabidopsis APETALA2 (AP2) which has two AP2 domains like SUI4/SNB binds ‘TTTGTT’ motif and the presence of this motif in the *AGAMOUS (AG)* second intron is important for the restriction of AG expression in vivo (Dinh et al. 2012). We investigated the presence of ‘TTTGTT’ motif in 2-kb upstream promoter and genic region of the five representative differentially expressed genes which are*, GIBBERELLIN 2-OXIDASE 3, GIBBERELLIN 20-OXIDASE 1, OsCKX 9*, *ONION 1,* and *OsPHI-1* (Additional file 8: Figure S5). All of these genes have ‘TTTGTT’ motifs in promoter region, which indicates possibility of direct binding of SUI4/SNB to the promoter region. However, further molecular study is required to determine whether these genes are direct targets of *SUI4/SNB.*

Originally, *SUI4/SNB* was identified as regulator of flower development (Lee et al. 2007). In contrast to phenotypes induced by knockout mutation, *sui4* mutants were able to maintain the native promoter-driven expression pattern by removing the suppression mechanism of microRNA. Instead of the production of multiple rudimentary glumes seen in *SNB* knockout plants (Lee et al. 2007), *sui4* mutants produced one more lemma and one less sterile lemma than wild-type plants.

The most valuable contribution of the present study is the evidence that the microRNA172 regulatory system is involved in the genetic network of internode elongation, which is developmentally controlled by phytohormonal action. The suppression of *SNB* by microRNA172 has already been well characterized (Lee et al. 2007; Lee et al. 2010; Zhu and Helliwell 2011). The present study showed that *SUI4/SNB* and microRNA172 play important roles not only in internode elongation but also in floral development. Since elongation of UPIs occurs concomitantly with panicle development, it has been suggested that signaling communication might take place between panicles and UPIs during the reproductive phase (Itoh et al. 2005; Wang et al. 2009; Yamamuro et al. 2000); the present study supports possible signaling pathways that mediate coincidence of panicle development and upper internode elongation. In both dicotyledons and monocotyledons, the expression level of microRNA172 increases as plants grow (Zhu and Helliwell 2011). Furthermore, mature microRNA172 accumulation increased significantly in leaves but not in roots as plants grew, reaching a maximum in the flag leaf (Zhu et al. 2009). It is possible that *SNB* suppresses internode elongation during the vegetative stage, and that this suppression ceases as microRNA172 increases in the culm during the reproductive stage. Further detailed study of the relationship between *SUI4/SNB* and microRNA172 expression during rice culm development should provide further insight into the possible *SUI4/SNB* and microRNA172 regulatory circuit controlling coincident development of panicles and upper internodes.

## Conclusion

Due to a mutation at the *microRNA172* binding site in *sui4* mutants, the expression of *SUI4*/*SNB* was enhanced, which lowered the activities of cell expansion and cell wall synthesis and consequently resulted in shortened internodes. This indicates that interaction between *SUI4*/*SNB* and microRNA172 could determine internode elongation during the reproductive stage in rice plants.

## Materials and methods

### Plant materials

Dongjin and *sui4* mutant *Oryza sativa* seeds were sown and the seedlings were grown in a greenhouse for one month before being transplanted into a paddy field. Two- and four-week-old seedlings were taken from the greenhouse and 70-day-old plants were taken from the field and RNA was extracted from the leaves. Additionally, RNA was extracted from the leaves, culms, and panicles of plants grown in the field in the booting and heading stages. At every stage, RNA was extracted from three independent plants. Five plants in the maturing stage were taken for measurement of internode lengths. The middle parts of the uppermost internodes in maturing stage plants were cut and fixed using 10% neutral buffered formalin solution. Then, these samples were embedded in paraffin, sectioned using a microtome, stained with hematoxylin, and observed using an AXIO Imager M1 microscope (ZEISS, Overkochen, Germany). With the photographs, the lengths of cells in parenchyma tissue of the uppermost internode were measured. The cells in three samples were measured for Dongjin plants and *sui4* mutants, respectively.

### Resequencing

The genomic DNA of *sui4* mutant plants was extracted using a DNeasy Plant Maxi Kit (QIAGEN, Hilden, Germany) and used for the preparation of sequencing libraries following the manufacturer’s protocols (Illumina, San Diego, USA). Fragments of the libraries were paired-end sequenced using a HiSeq™ 2000 platform (Illumina, San Diego, USA). The genome sequence data of Dongjin produced using a HiSeq™ 2000 platform was downloaded from Sequence Read Archive (SRA) database of NCBI (https://www.ncbi.nlm.nih.gov/sra): Their accession numbers were ERR157946 and ERR157947. The length of all sequences generated was 101 nucleotides. High-quality raw reads, based on Phred quality values > Q20, were used to analyze genetic variation. The genetic sequence of *Oryza sativa* L. cv. Nipponbare was used as a reference (Pseudomolecules IRGSP-1.0, https://rapdb.dna.affrc.go.jp/download/irgsp1.html, Rice Genome Sequencing Project 2008). The CLC Assembly Cell program (ver. 3.2.2, http://www.clcbio.com) was used for read mapping and SNP detection.

### Candidate gene isolation and transformation

The full *Os07g0235800* genomic region from Dongjin plants was amplified by a primer pair of 5′- CGGTACCCGGGGATCCAAATCAGTTCCTACGTGCAACGGGCGCAAAATATC-3′ and 5′- CGACTCTAGAGGATCCGCCCCACTTCCTTGGGAAACTTAAACGATGAGCTC-3′ using PrimeSTAR GXL Polymerase (TaKaRa, Shiga, Japan). The PCR products were purified by gel elution and introduced into a pCAMBIA1300 vector using an In-Fusion HD Cloning Kit (Clontech, Mountain View, USA). The transformation vector thus produced was sequence-verified by sequencing the introduced gene, then used for transformation of *sui4* mutants. The full *Os07g0235800* genomic region from *sui4* mutants was amplified and introduced into the pCAMBIA1300 vector in the same way. The transformation vector thus produced was used for transformation of Dongjin plants. Site-directed mutagenesis was performed with the aforementioned *sui4* transformation vector using a QuickChange II XL Site-Directed Mutagenesis Kit (Agilent Technologies, Santa Clara, USA) according to the instruction manual. A primer pair consisting of 5′- CCATTACTCCCTACTGCAGCAAGTTCAGGATTCTCTACGG-3′ and 5′- CCGTAGAGAATCCTGAACTTGCTGCAGTAGGGAGTAATGG-3′ was used for mutagenesis. The vector thus produced was used for transformation of Dongjin plants. To make a fusion construct between the GUS reporter gene and *Os07g0235800* 2-kb promoter, the promoter region was amplified first by a primer pair of 5′- AAAAAGCAGGCTAAATCAGTTCCTACGTGCAACGGGCGCAAAATATC-3′ and 5′- AGAAAGCTGGGTACTAACCAACCGCTCTCCCCCGCGC-3′ and then with primers attB1 (5′-GGGGACAAGTTTGTACAAAAAAGCAGGCT-3′) and attB2 (5′-GGGGACCACTTTGTACAAGAAAGCTGGGT-3′). The resulting attB-PCR products were cloned by a BP clonase reaction (Invitrogen, Calsbad, USA) into the Gateway pDONR 201 cloning vector (Invitrogen). Subsequently, we used an LR clonase (Invitrogen) reaction to insert the fragment into the destination vector, pMDC163. The vector thus produced was used for transformation of Dongjin plants. Once per week during the month before the heading stage, when panicle development and internode elongation occurred, culms of transgenic plants were taken and the GUS expression pattern was observed. Histochemical GUS staining of transformants was performed according to the protocol described by Dai et al. (1996).

### RNA-seq analysis and qRT-PCR

The main culms of *sui4* mutant plants and wild-type Dongjin plants were taken at the booting stage. RNA was extracted from culms using an RNeasy Plant Mini Kit (QIAGEN). Three RNA samples from three main culms were used for RNA-seq analysis for *sui4* mutant and Dongjin plants, respectively. Total RNA quality and quantity was verified using a NanoDrop1000 spectrometer (Thermo Scientific, Wilmington, USA) and Bioanalyzer 2100 (Agilent technologies). We used TruSeq RNA Library Preparation Kit (Illumina) for sequencing library preparation. The TruSeq library was sequenced using the HiSeq™ 2000 platform (Illumina). The RNA-seq reads were mapped to the *Oryza sativa* reference genome using TopHat (Trapnell et al. 2009). The transcript counts were calculated and the relative transcript abundances were measured in FPKM (fragments per kilobase of exon per million fragments mapped) using Cufflinks (Trapnell et al. 2010). For each transcript, we conducted independent t-tests between *sui4* mutant and Dongjin plants. Finally, we determined the DEGs by adjusting |fold change| ≥ 2 and independent t-test raw *p* < 0.05. Biological gene functional annotation analysis was performed for the DEG list using the DAVID tool (http://david.abcc.ncifcrf.gov/) (Huang et al. 2007) to understand the biological meanings behind the list of DEGs. In the DAVID annotation system, modified Fisher’s exact *p*-values (EASE scores) are adopted to measure gene enrichment in annotation terms. The EASE score was corrected using the Benjamini method to reduce false positives caused by multiple testing. All GO terms < 0.05 after Benjamini correction were taken as significantly enriched GO terms.

qRT-PCR analysis for the representative DEGs was performed using the RNA used for RNA-seq. cDNA was synthesized using a PrimeScript 1st strand cDNA Synthesis Kit (Takara). Primers, shown in Additional file 3: Table S3, were designed on the GenScript Real-time PCR Primer Design website (https://www.genscript.com/ssl-bin/app/primer). The rice ubiquitin 1 gene was used as a reference gene for normalization. PCR amplifications were performed in 20 μl final volumes containing AccuPower 2X GreenStar qPCR MasterMix (BiONEER, Daejeon, Korea), 10 pmol of each primer, and 2 μl cDNA solution on a CFX96 Real-Time PCR Detection System (Bio-Rad, Hercules, USA). Three biological replicates were performed. Relative gene expression was calculated by Bio-Rad CFX Manager 3.1 software (Bio-Rad).

### Accession codes

The sequencing data of *sui4* mutant have been submitted to NCBI (https://www.ncbi.nlm.nih.gov/) under the accession number of SRR7942370 and SRR7942369. The accession numbers of the sequencing data of Dongjin were ERR157946 and ERR157947.

## Additional files

Additional file 1: **Table S1.** List of SNPs in the *SUI4* mapped region of Dongjin plants and *sui4* mutants. (DOCX 16 kb)

Additional file 2: **Table S2.** Differentially expressed genes (at least two fold change in expression, p-value< 0.05) in culm (XLSX 55 kb)

Additional file 3: **Table S3.** qRT-PCR primer sequences (DOCX 14 kb)

Additional file 4: **Figure S1.** RT-PCR of candidate genes for the *sui4* mutant phenotype. Expression of the five genes with SNPs between Dongjin and *sui4* mutant in the mapped region was measured by RT-PCR. (JPG 20 kb)

Additional file 5: **Figure S2.** Culm structure of *Os07g0235800* transgenic plants. Arrows indicate locations of nodes. Two main culms from two plants are shown for each phenotype. Two independent transgenic lines are shown for *SUI4-DJ* and *DJ-SUI4*. (JPG 49 kb)

Additional file 6: **Figure S3.** Flower structure of an *sui4* mutant and a transgenic line. (A), (B): wild type, (C), (D): *sui4* mutant, (E), (F): *DJ-SUI4* transgenic line. rg: rudimentary glume, sl: sterile lemma, le: lemma, pa: palea (JPG 73 kb)

Additional file 7: **Figure S4**. *Os07g0235800* gene expression in Dongjin, *sui4*, DJ-SUI4, and DJ-SUI4sd plants. Relative fold expression difference is based on the expression level detected in 14 day old seedling leaves of Dongjin. Error bars represent standard deviation of the expression ratio. 14 L: 14 day old seedling leaves, 28 L: 28 day old seedling leaves, 77 L: 77 day old plant leaves, DJ: Dongjin, DJ-SUI4: transgenic line in which the SUI4 gene from a *sui4* mutant was introduced into Dongjin plants, DJ-SUI4sd: transgenic line in which the SUI4 gene, modified by site-directed mutagenesis as shown in Fig. 3a, was introduced into Dongjin plants. (JPG 42 kb)

Additional file 8: **Figure S5**. Location of AP2 domain binding motif ‘TTTGTT’ in promoter and genic regions in the five representative genes which are differentially expressed between Dongjin plants and sui4 mutants. The locations of ‘TTTGTT’ motif are indicated by brown vertical lines. Filled gray boxes indicate 5′ and 3′ UTRs, and filled black boxes indicate exons, including protein-coding sequence. Gray lines indicate introns, and green lines indicate 2-kb upstream promoter region. (JPG 39 kb)

## Data Availability

The datasets supporting the conclusions of this article are included within the article and its additional files. The accession codes of the resequencing data of *sui4* mutant and Dongjin were indicated.

## Abbreviations

BRs: Brassinosteroids
*BZR1*: *BRASSINAZOLE-RESISTANT 1*
DEGs: differentially expressed genes
DZ: differentiation zone
*Ehd1*: *EARLY HEADING DATE 1*
EZ: elongation zone
FPKM: fragments per kilobase of exon per million fragments mapped
GA: Gibberellin
GO: Gene ontology
GUS: β-glucuronidase
*IBH1*: *ILI1 BINDING BHLH 1*
*ILI1*: *INCREASED LAMINA INCLINATION 1*
IM: intercalary meristem
miR172: microRNA172
*ONI1*: *ONION1*
*OsAP2–39*: *Oryza sativa APETALA2–39*
*OsCKX 9*: *Oryza sativa CYTOKININ OXIDASE 9*
*OsGA20OX1*: *Oryza sativa GIBBERELLIN 20-OXIDASE 1*
*OsGA2OX3*: *Oryza sativa GIBBERELLIN 2-OXIDASE 3*
*OsIDS1*: *Oryza sativa INDETERMINATE SPIKELET1*
*PRE1*: *PACLOBUTRAZOL RESISTANCE 1*
qRTPCR: quantitative reverse transcription-PCR
*SLR 1*: *SLENDER RICE1*
*SNB*: S*UPERNUMERARY BRACT*
*sui4*: *shortened uppermost internode 4*
UPIs: Upper internodes
