# Supplementary material for: Mutations in the microRNA172 binding site of SUPERNUMERARY BRACT (SNB) suppress internode elongation in rice
Source: Rice (N Y). 2019 Aug 9;12:62. doi: 10.1186/s12284-019-0324-8 (PMC6689044; doi:10.1186/s12284-019-0324-8)
Supplement: Supplementary file 1 — Table S1. List of SNPs in the SUI4 mapped region of Dongjin plants and sui4 mutants. (DOCX 16 kb) [file 12284_2019_324_MOESM1_ESM.docx]

**Table S1.** List of SNPs in the *SUI4* mapped region of Dongjin plants and *sui4* mutants.

| **Chromosome** | **Position** | **Reference sequence nucleotide** | **Dongjin allele** | ***sui4* allele** | **Location** |
| --- | --- | --- | --- | --- | --- |
|  |  |  |  |  |  |
| chr07 | 7,001,956 | G | G | T | Intergenic |
| chr07 | 7,001,959 | T | T | A | Intergenic |
| chr07 | 7,265,976 | G | G | T | *Os07g0229900* intron |
| chr07 | 7,285,032 | C | C | A | Intergenic |
| chr07 | 7,309,292 | C | C | A | Intergenic |
| chr07 | 7,328,504 | A | A | T | Intergenic |
| chr07 | 7,367,868 | T | T | - | Intergenic |
| chr07 | 7,377,677 | A | A | G | Intergenic |
| chr07 | 7,381,992 | G | G | - | *Os07g0232200* intron |
| chr07 | 7,471,002 | G | G | A | Intergenic |
| chr07 | 7,567,079 | - | - | T | Intergenic |
| chr07 | 7,568,170 | - | - | A | Intergenic |
| chr07 | 7,581,480 | T | T | A | *Os07g0235800* CDS ^a^ |
| chr07 | 7,597,462 | G | G | T | Intergenic |
| chr07 | 7,597,463 | A | A | T | Intergenic |
| chr07 | 7,660,336 | T | T | A | *Os07g0237100* intron |
| chr07 | 7,680,581 | T | T | A | Intergenic |
| chr07 | 7,696,080 | T | T | A | Intergenic |
| chr07 | 7,818,571 | A | A | G | Intergenic |
| chr07 | 7,825,151 | C | C | A | *Os07g0240300* intron |
| chr07 | 7,825,152 | A | A | T | *Os07g0240300* intron |
| chr07 | 8,099,960 | A | A | T | Intergenic |

^a^ This SNP causes amino acid substitution from serine to threonine.
