# Supplementary material for: Mutations in the microRNA172 binding site of SUPERNUMERARY BRACT (SNB) suppress internode elongation in rice
Source: Rice (N Y). 2019 Aug 9;12:62. doi: 10.1186/s12284-019-0324-8 (PMC6689044; doi:10.1186/s12284-019-0324-8)
Supplement: Supplementary file 3 — Table S3. qRT-PCR primer sequences (DOCX 14 kb) [file 12284_2019_324_MOESM3_ESM.docx]

**Table S3**. qRT-PCR primer sequences

| Gene | Forward Primer | Reverse Primer |
| --- | --- | --- |
| *SUI4/SNB* | ATGGAAGGGAAGCTGTTAC | AATGTGGATGCTGGGACATC |
| *GA 2-OXIDASE 3 (Os01g0757200)* | GTTGCAGGTTCTGACCAATG | GGTGCAATCCTCTGTGCTAA |
| *GA 20-OXIDASE 1 (Os03g0856700)* | CGAGGGTGTACCCGGACTTC | AGTCGGAGAAGGCCTGAAGC |
| *OsCKX9 (Os05g0374200)* | CACACCAGAAGAGCCATGAG | GAGTTCCTCCAGGGATGATG |
| *ONION1 (Os03g0181500)* | GACGAACTCAAGGTGTCGAA | GCGGCATGTAGGACTCGT |
| *DIHYDROSPHINGOSINE C4 HYDROXYLASE 1 (Os06g0226950)* | TGCTTCCTCCTCCTGTGTGT | AGCCTGTAGTCGTCCATCCC |
| *Os12g0104400* | GCACCCGATCACCGAGAAAT | TGCCCTGGGACATGTTCATC |
| *Os01g0134500* | TTGCAAGCCATACCGCATGT | AGTTGTCCACACGGCCTCTA |
| *OsPHI-1 (Os02g0757100)* | CTTCTCTTCCAGATGTGCGT | CCCTTGTGGTACGTCATTGT |
| *Os07g0529700* | GGTATCTCTTCGGGTGGTTC | TCGGAGCACATGTAGTAGGC |
| *Os01g0842400* | ACCATGGCTATCTCCTCTGC | CATTTGCACATTGAATTCGTAG |
| *Os04g0530100* | TCGGTCGTCTACAGAAGTGTTC | AACTCGACCAGCAGTGACAG |
| *Os09g0262000* | TATCTGTTGATCCGCGACTT | GCTCCTATTTGGGTCCATGT |
| *Ubiquitin 1* | AACCAGCTGAGGCCCAAGA | ACGATTGATTTAACCAGTCCATGA |
