# Supplementary figures and images for: Mutations in the microRNA172 binding site of SUPERNUMERARY BRACT (SNB) suppress internode elongation in rice
Source: Rice (N Y). 2019 Aug 9;12:62. doi: 10.1186/s12284-019-0324-8 (PMC6689044; doi:10.1186/s12284-019-0324-8)

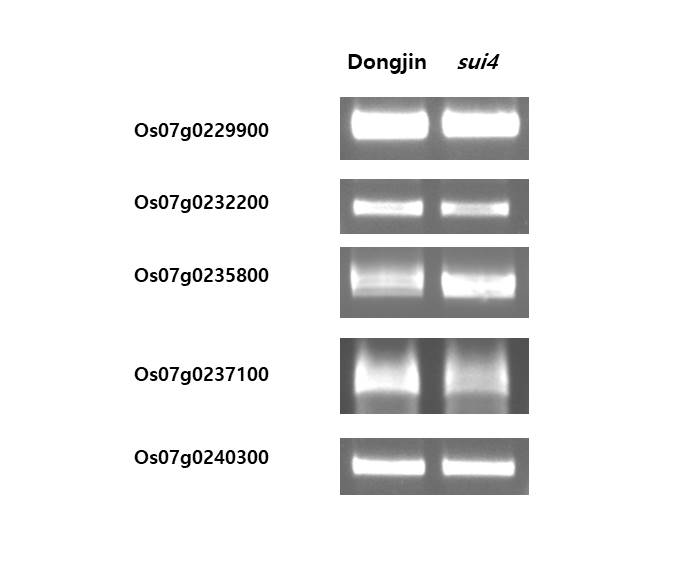

Supplement: Supplementary file 4 — Figure S1. RT-PCR of candidate genes for the sui4 mutant phenotype. Expression of the five genes with SNPs between Dongjin and sui4 mutant in the mapped region was measured by RT-PCR. (JPG 20 kb) [file 12284_2019_324_MOESM4_ESM.jpg]

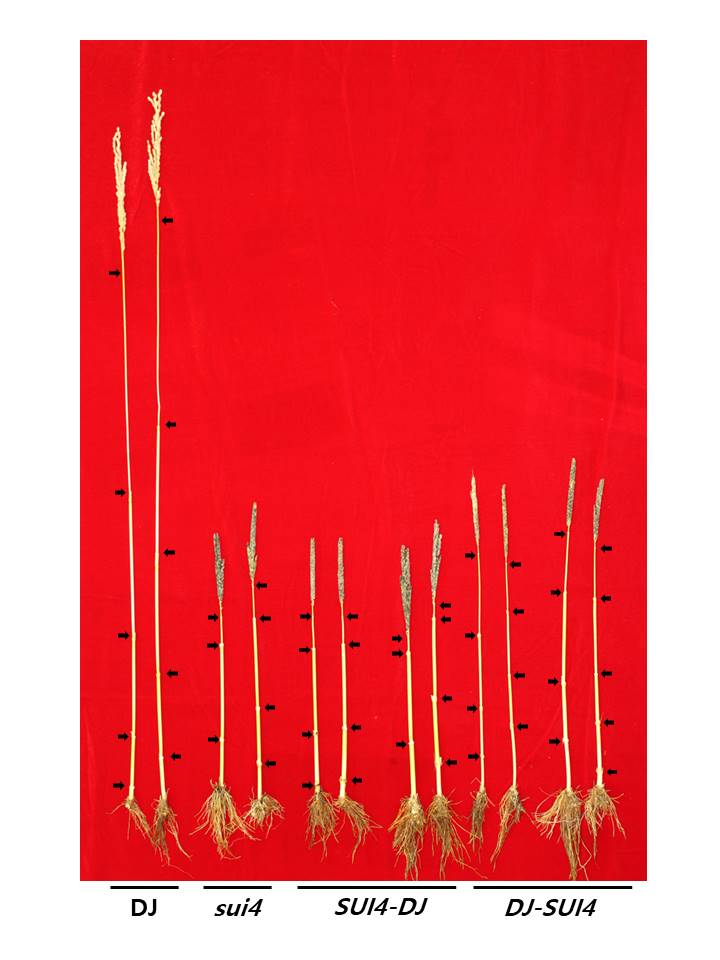

Supplement: Supplementary file 5 — Figure S2. Culm structure of Os07g0235800 transgenic plants. Arrows indicate locations of nodes. Two main culms from two plants are shown for each phenotype. Two independent transgenic lines are shown for SUI4-DJ and DJ-SUI4. (JPG 49 kb) [file 12284_2019_324_MOESM5_ESM.jpg]

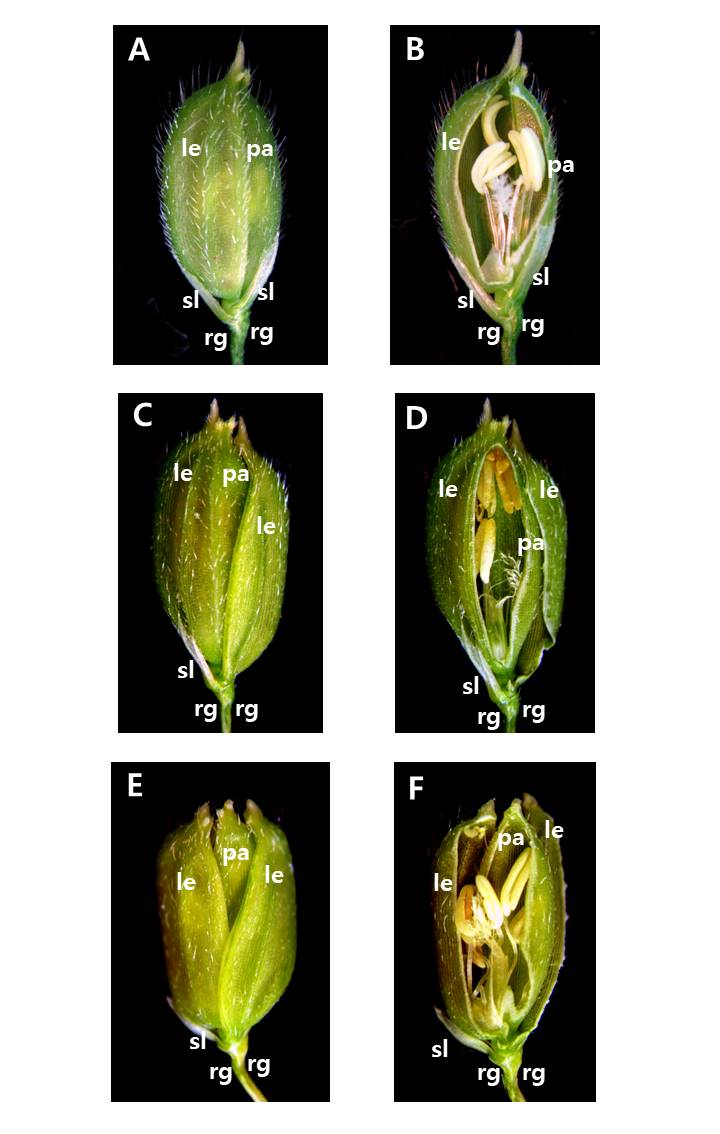

Supplement: Supplementary file 6 — Figure S3. Flower structure of an sui4 mutant and a transgenic line. (A), (B): wild type, (C), (D): sui4 mutant, (E), (F): DJ-SUI4 transgenic line. rg: rudimentary glume, sl: sterile lemma, le: lemma, pa: palea (JPG 73 kb) [file 12284_2019_324_MOESM6_ESM.jpg]

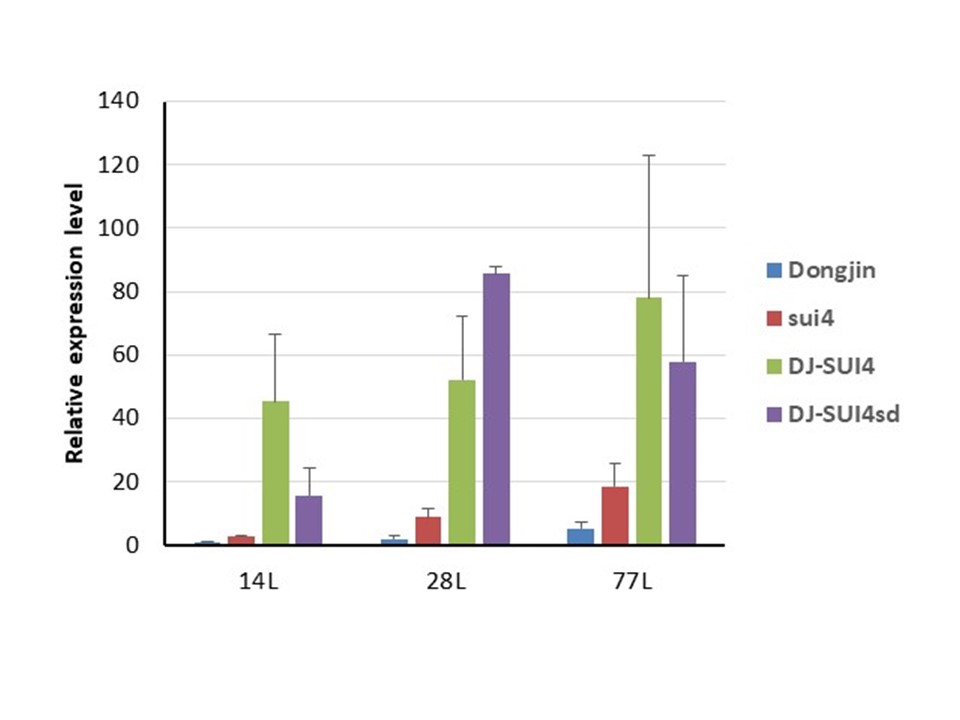

Supplement: Supplementary file 7 — Figure S4. Os07g0235800 gene expression in Dongjin, sui4, DJ-SUI4, and DJ-SUI4sd plants. Relative fold expression difference is based on the expression level detected in 14 day old seedling leaves of Dongjin. Error bars represent standard deviation of the expression ratio. 14 L: 14 day old seedling leaves, 28 L: 28 day old seedling leaves, 77 L: 77 day old plant leaves, DJ: Dongjin, DJ-SUI4: transgenic line in which the SUI4 gene from a sui4 mutant was introduced into Dongjin plants, DJ-SUI4sd: transgenic line in which the SUI4 gene, modified by site-directed mutagenesis as shown in Fig. 3a, was introduced into Dongjin plants. (JPG 42 kb) [file 12284_2019_324_MOESM7_ESM.jpg]

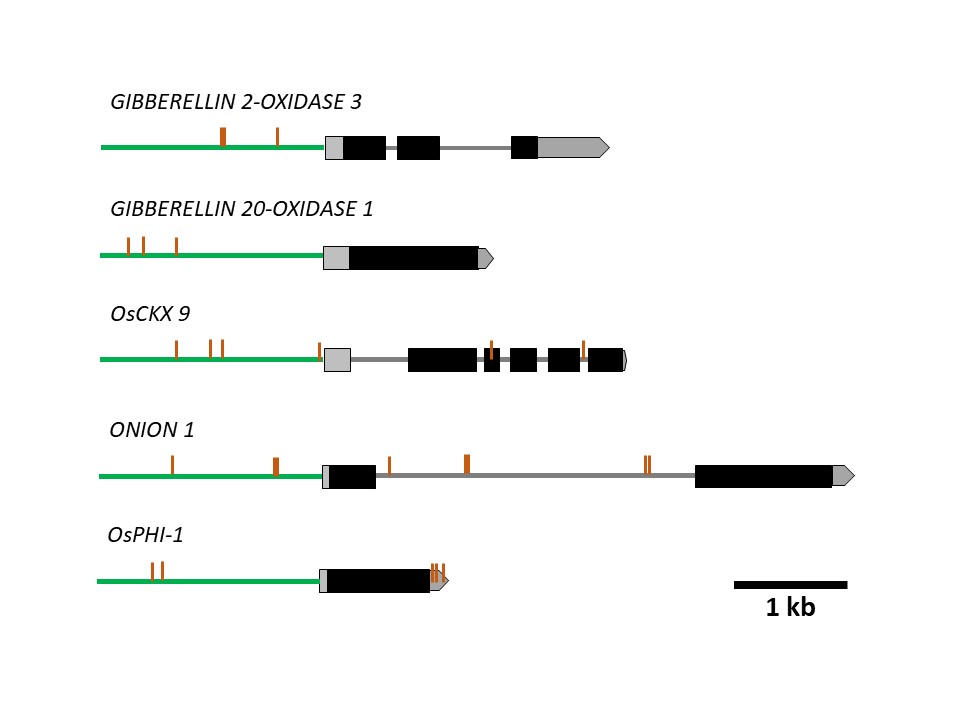

Supplement: Supplementary file 8 — Figure S5. Location of AP2 domain binding motif ‘TTTGTT’ in promoter and genic regions in the five representative genes which are differentially expressed between Dongjin plants and sui4 mutants. The locations of ‘TTTGTT’ motif are indicated by brown vertical lines. Filled gray boxes indicate 5′ and 3′ UTRs, and filled black boxes indicate exons, including protein-coding sequence. Gray lines indicate introns, and green lines indicate 2-kb upstream promoter region. (JPG 39 kb) [file 12284_2019_324_MOESM8_ESM.jpg]
